# Multimorbidity and consultation time: a systematic review

**DOI:** 10.1186/s12875-020-01219-5

**Published:** 2020-07-28

**Authors:** Ana Carolina Reis Tadeu, Inês Rosendo Carvalho e Silva Caetano, Inês Jorge de Figueiredo, Luiz Miguel Santiago

**Affiliations:** 1 Faculty of Medicine, University of Coimbra, Coimbra, Portugal; 2 USF Coimbra Centro, Coimbra, Portugal; 3 ACeS Dão Lafões, Coimbra, Portugal; 4 Faculty of Health Sciences, University of Beira Interior, Covilhã, Portugal; 5 General Practice/Family Practice clinic of the Faculty of Medicine of University of Coimbra, Coimbra, Portugal; 6 Center for Health and Investigation studies of the University of Coimbra (CEISUC), Coimbra, Portugal

**Keywords:** Multimorbidity, Medical appointment, Quality of healthcare, Consultation length

## Abstract

**Background:**

Multimorbidity (MM) is one of the major challenges health systems currently face. Management of time length of a medical consultation with a patient with MM is a matter of concern for doctors.

**Methods:**

A systematic review was performed to describe the impact of MM on the average time of a medical consultation considering the Preferred Reporting Items for Systematic Review and Meta-analyses (PRISMA) guidelines. The systematic online searches of the Embase and PubMed databases were undertaken, from January 2000 to August 2018. The studies were independently screened by two reviewers to decide which ones met the inclusion criteria. (Kappa = 0.84 and Kappa = 0.82). Differing opinions were solved by a third person. This systematic review included people with MM criteria as participants (two or more chronic conditions in the same individual). The type of outcome included was explicitly defined – the length of medical appointments with patients with MM. Any strategies aiming to analyse the impact of MM on the average consultation time were considered. The length of time of medical appointment for patients without MM was the comparator criteria. Experimental and observational studies were included.

**Results:**

Of 85 articles identified, only 1 observational study was included, showing a clear trend for patients with MM to have longer consultations than patients without MM criteria (*p* < 0.001).

**Conclusions:**

More studies are required to better assess allocation length-time for patients with MM and to measure other characteristics like doctors’ workload.

## Background

Multimorbidity (MM) is defined by the European General Practice Research Network as “any combination of chronic disease with at least one other disease (acute or chronic) or biopsychosocial factor (associated or not) or somatic risk factor” [1]. This is sometimes simplified to, “the simultaneous occurrence of two or more chronic diseases in the same individual” [2]. MM is now one of the main challenges faced by health systems at an international level and occupies a considerable part of the daily activity of General Practitioners/Family Doctors (GPs/FMs) around the world [3–6].

With an ever-ageing world population, MM and its consequences, are becoming a major issue in public health and primary care. According to United Nations data [7, 8], Europe has the largest percentage of population aged 60 or over (25%) [7]. In 2015 the number of people in the world aged 60 years and older was 901 million [8]. It is projected that in 2030 this figure will rise to 1.4 billion (a 56% increase since 2015) and stand at 2.1 billion in 2050 [8]. Several studies have shown that there is a significant association between age [2] and the prevalence of MM, most national health systems not being prepared or able to cope with this rapid ageing with many demands [5, 6]. MM reduces life expectancy and quality of life (QoL) [5]. However, QoL can increase if the quality of care (QoC) improves [9], and this can require additional consultation time.

So, it is imperative to think about the most correct approach to patients with MM in order to maximize the QoC provided by the Health Services (HS), and so ensure a better quality of life.

GPs/FMs medical team face various difficulties in caring for a patient with MM like lack of resources; consultation time restrictions [10]; lack of interdisciplinary care/teams; inadequate patient support (largely relying on community-based support services); inadequate tools (guidelines are drawn up strictly for specific diseases and not for the MM patient); the attitude of the patient (often discouraged and poorly engaged) [4, 11].

Information about the length-time of a consultation with a patient with MM is essential to better organize and deliver healthcare. To our knowledge, no previous review has summarized data related to: What is the impact of having MM on the medical consultation?; and Is the average length-time consultation with a patient with MM longer than for a patient without MM?.

## Methods

A systematic review was performed considering the Preferred Reporting Items for Systematic Review and Meta-Analyses (PRISMA) guidelines for systematic reviews and meta-analysis, not following any review protocol. This research proposal has been submitted to and approved by the Faculty of Medicine at the University of Coimbra.

### Eligibility criteria

The PICO model has been followed to define the question and allow the most effective literature search. The population (P) was defined as people with MM. The most widely used definition of MM was used, which is the coexistence of two or more chronic conditions in the same individual [2]. The World Health Organization (WHO) definition of chronic disease was adopted, namely, health problems that require ongoing care over a long period of time (years or decades) [12].

As interventions (I) any strategies aiming to analyse the impact of MM on the average consultation time were considered. The length of time of medical appointment for patients without MM was the comparator criterion (C). As outcome (O), it was expected to explicitly define the length-time of medical appointments with patients with MM. Experimental and observational studies were included. Studies which did not specify the time spent on medical appointments were excluded.

### Information sources and search strategy

The systematic online searches were implemented using combinations of keywords in the following electronic databases: the Embase and PubMed databases, from January 1, 2000 until August 31, 2018, an 18-year time period, to find pertinent studies. The authors believe that the most reliable way to assess consultation time is through computerized clinical records, which were very scarce before 2000.

The search within the Embase database used the following combination of keywords: (‘multiple chronic conditions’/exp. OR’multiple chronic conditions’) AND (‘consultation time’ OR ((‘consultation’/exp. OR consultation) AND (‘time’/exp. OR time))); (‘multiple chronic conditions’/exp. OR ‘multiple chronic conditions’) AND (‘primary health care’/exp. OR ‘primary health care’) AND (‘time’/exp. OR time); ‘consultation’/exp. AND ‘multiple chronic conditions’/exp./mj; ‘multiple chronic conditions’/exp. AND (‘time’/exp. OR’average’/exp. OR ‘consultation’/exp). For the PubMed database the combinations of keywords were: “Chronic Disease/epidemiology”[Mesh] AND ((“referral and consultation”[MeSH Terms] OR (“referral”[All Fields] AND “consultation”[All Fields]) OR “referral and consultation”[All Fields] OR “consultation”[All Fields]) AND (“time”[MeSH Terms] OR “time”[All Fields])).

The search was limited to papers in English, Portuguese, Spanish and French published in internationally-recognized peer-reviewed journals to ensure the reliability of the data. No other limits were imposed during this stage of the study. References in the identified literature were scanned for further literature when it was found appropriate to support work decisions.

### Data extraction and quality assessment

The potentially relevant studies containing quantitative consultation time data were selected in two stages. First, the titles and abstracts quoted in the literature search were independently screened by two reviewers (the first and third co-authors of this work) to decide which ones to accept, meeting the inclusion criteria (Kappa = 0.84). Those not meeting the inclusion criteria were excluded. Differing opinions on a study’s inclusion were resolved by a third person (the second co-author of this work).

Secondly, the researchers independently read and analysed the integrity of the matching studies and tried to reach an agreement concerning eligibility (Kappa = 0.82). Those not meeting the inclusion criteria were excluded. Differing opinions on a study’s inclusion were resolved by a third person (the second co-author of this work). The quality and risk of bias of the included studies was assessed using the Newcastle-Ottawa Scale (NOS), more precisely, the Newcastle-Ottawa scale adapted for cross-sectional studies [13]. The NOS is widely used and recommended by the Cochrane Collaboration [14], and the authors found it suitable for the purpose of this work. This tool assesses three aspects of a study: the selection of the sample; the comparability of the groups; and the outcome (assessment of outcome and statistical test). It is composed of 7 items and classifies the study in 4 possible levels: Very good (9–10 points), Good (7–8 points), Satisfactory (5–6 points) and Unsatisfactory (0–4 points). Any disagreement was resolved through consensus.

This systematic review was conducted using Covidence 13, the standard production platform used for Cochrane reviews, which was used for the data and records management.

### Outcomes and statistical analysis

The patients were split into two groups, those with and those without MM, and the relative frequencies were calculated. The results were analysed using the chi-square distribution test.

## Results

### Study selection

**Fig. 1:**
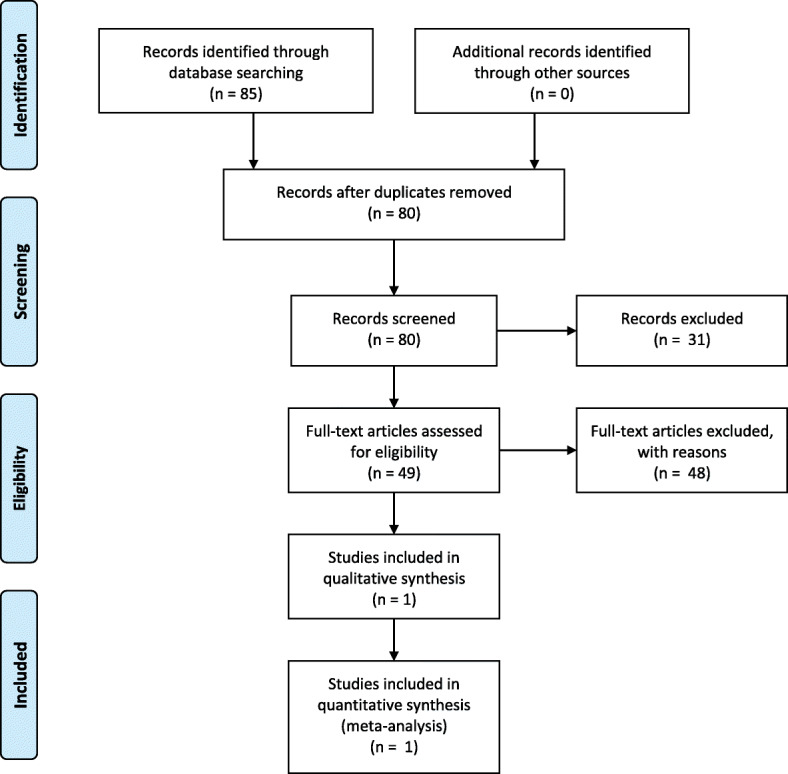
PRISMA Flow Diagram [15]– Literature search and selection process for studies included

As described in Fig. 1, the electronic database searches started with 85 potentially eligible references (16 in PubMed and 59 in Embase). Of these, 5 were duplicates and were thus excluded and 31 were considered irrelevant based on a review of the title and abstract. The remaining studies were fully read, analysed and assessed for eligibility; 36 were then excluded due to unsatisfactory outcome [3, 16–49], 5 due to unsuitable study design [50–54], 4 due to unsuitable patient population [11, 55–57], 2 due to inadequate language [58, 59] and 1 to unsatisfactory setting [60]. In the end, 1 study was included [61].

### Study characteristics and quality

The main relevant features and outputs of the study were extracted for the purpose of this systematic review and are summarized in Table 1.

**Table 1:** Summary of study’s characteristics

Author	Year of study	Country	Design	Number of participants	Population (inclusion criteria)	Setting	Method of data collection	Outcomes measured	Author’s conclusions
Moth et al	2008–2009	Denmark	Cross-sectional	404 GPs, 8236 contacts	Persons aged 40 years or more	General practice	Registration form completed by GP about all patient contacts on one randomly assigned date during the study period.	- Length of consultation time, chronic disease, reason for appointment, diagnosis, number of additional psychosocial problems raised by the patient during the consultation, difficulty found with consultation of the consultation, referral to specialized care, and whether a nurse could have replaced the GP.	- GPs found consultations with patients suffering from chronic conditions to be more difficult than those with patients without chronic disease (*p* < 0.001).

*GP* General Practitioner

The included study was conducted between 2008 and 2009 in Denmark, over 12 months. It involved 404 general practitioners (GPs) participants and a total of 8236 contacts. It included patients aged 40 years or more, grouped as those without any chronic condition and those with one, two, three or more chronic conditions.

During the study period, the GPs completed a one-page registration form for each of their patient contacts. Of the various items that were registered, the ones relevant for this review were information on chronic diseases and length-time of consultation. Quality assessment result, performed as described in methodology, is presented in Table 2. The quality of the study was considered satisfactory (score 6 out of a maximum score of 10), its main weakness being in the comparability section.

**Table 2:** Quality of study - Newcastle-Ottawa Scale adapted for cross-sectional studies

Author	Selection				Comparability	Outcome		Total score	Power
	Representativeness of the sample	Sample size	Non-respondents	Ascertainment of exposure (risk factor)	Comparability of subjects in different outcome groups on the basis of design or analysis.	Assessment of outcome	Statistical test		
Moth et al	*	- (b)	*	**	- (c)	*	*	6	Satisfactory

* - score of 1 point; ** - score of 2 points; (b) – Calculation not reported; (c) – Data/results not adjusted for all relevant confounders not provided

Cross-sectional Studies: Very Good Studies (9–10 points); Good Studies (7–8 points); Satisfactory Studies (5–6 points); Unsatisfactory Studies (0 to 4 points)

### Results of study

Table 3 shows the relationship between the length of consultation time and the type of patient (with and without MM). There is a clear significant trend for patients with MM to have longer consultations than patients without MM (*p <* 0.001).

**Table 3:** Length of consultation time and type of patient (with and without MM criteria)

Length of consultation time	No MM (n) %		MM (n) %		*p*-value
< 5 min	293	11.7	96	7.7	*p* < 0.001
6–15 min	1686	67.3	804	64.9	
16–30 min	485	19.4	314	25.3	
> 30 min	42	1.7	25	2.0	
Total	2506	100	1239	100	

More than 25% of MM patients had a consultation length-time of at least 16 min while more than 75% of the patients without MM had a consultation length-time under 15 min. Length-time consultation on both types of patients is more frequently between 6 and 15 min. There is a significant difference, however, in the percentage of patients with MM requiring more time than patients without MM criteria.

## Discussion

### Main findings

The present systematic review sought to find out if the average consultation time spent on patients with MM is longer than that spent on patients who do not meet the MM criteria. It could only identify one study [61], undertaken in Denmark, in which the consultation time was logged as a function of the number of chronic diseases. This study revealed a tendency for consultations to take longer for patients with MM than for those without MM. However, the study was not directly aimed at answering this question and it did not take confounding factors into account. In addition, it does not describe the calculation to determine the sample size of the study and it could be inaccurate to study this specific outcome still it answers the posed question.

### Comparison with existing literature

The small number of publications in the literature on this subject shows that more studies should be designed to investigate the impact of MM on the consultation length-time. It is vital to analyse this issue in order to manage resources so that they meet the actual need, and to ensure the services provided by health services, national or private, are appropriate. It will thus be possible to guarantee better quality health services and outcomes for these patients. In fact, MM is about a patient with more than two chronic diseases or one chronic disease with biopsychosocial factor (associated or not) or somatic risk factor, so illness being included and not only about the specific sufferances [1, 11].

In studies with calculated size population representative random samples it is important that accurate methods are used to measure the real length-time of a consultation using appropriate tools. Confounding factors must be eliminated as the time spent on administrative work. Only direct observation using video recording has been proven to obtain accurate values when measuring the duration of consultations [62], which could be a procedure that mitigates many of the errors previously mentioned. It is essential to identify beforehand any possible confounding factors inherent to the patients (for example, hearing difficulty, education level, age, socio-economic level), inherent to the doctor (in particular, a change in behaviour due to the participation in the research study – Hawthorne effect [63]), and inherent to the consultation/institution (for example, glitches in computer systems, organization of necessary information in the health informatics records, coding errors, telephone call interruptions). The time lost searching for information in consultation, the friendliness of clinical informatics and the time spent on records are also issues to be studied and thought of [64, 65]. Health determinants are factors to be studied in such a MM population for better health and social outcomes [66].

The data analysis must be evaluated using objective validated laboratory methods and, if possible, it should be a blind assessment. Statistical tests used to analyse the data must be appropriate and clearly described. Measures of association, including confidence intervals and the *P* value, must be presented.

### Further considerations

The QoL of MM patients can increase if the quality of QoC improves, so the improvement of the quality of services provided by the HS is an important matter.

Patients with MM require more medical resources and longer consultation length. Medical teams need to be interdisciplinary and guidelines drawn up for specific diseases need to be improved for MM patients.

HS need to provide good patient support and to acquire knowledge to deal with often discouraged and poorly engaged patients.

The appropriate length for a consultation of a patient with MM needs to be taken into account to better organize and deliver healthcare, taking into account that the frequency of consulting and the number of problems to discuss are significantly higher in the patients with MM [67].

### Strengths and limitations

The main limitation of this systematic review was the difficulty in ensuring that all the relevant literature was included. Even though the research used two of the main databases – PubMed and Embase – there could be other relevant material in grey literature, not taken into account in the present work to ensure the reliability of the data.

The scarcity of the literature that was found was a limitation for this review. The one publication found, besides not directly answering our question, also does not take confounding factors into account, and does not describe the calculation to determine the sample size of the study. However, it does highlight the relevance of the subject matter. The authors believe that the lack of recognition of MM and fragile patients in the clinical management of patients is also one of the reasons why scientific research has paid less attention to MM.

## Conclusions

This impact of MM on the duration of a consultation has hardly been studied, this systematic review shows.

A tendency for consultations of patients with MM to take longer than those without MM was found in the only one study with “satisfactory” quality which met the inclusion criteria.

So more research is needed to acquire more information on this subject, important to deal not only with diseases but with the person suffering from MM for consultation must have the adequate length duration to enable doctors and stake-holders with a proper quantification of the time and associated costs.

If a longer consultation time is confirmed, it will be important to rethink and adapt GPs’ lists of patients in order to achieve better medical care providing agendas with specific times and allocating enough time for all the required tasks.

## Data Availability

Materials can be assessed upon request.

## Abbreviations

MM: Multimorbidity
QoL: Quality of life
QoC: Quality of care
GP: General Practitioner
PRISMA: Preferred Reporting Items for Systematic Review and Meta-analyses
HS: Health Service
WHO: World Health Organization
